# Re-Imagining Healthcare and Medical Research Systems in Post-Devolution Scotland

**DOI:** 10.1177/1360780418823221

**Published:** 2019-03-11

**Authors:** James Mittra, Michele Mastroeni, Gill Haddow, David Wield, Elisabeth Barlow

**Affiliations:** University of Edinburgh, UK; OCAD University, Canada; University of Edinburgh, UK; The Open University, UK; University of Edinburgh, UK

**Keywords:** health policy, imagined community, medical research, referendum debate, Scottish devolution

## Abstract

We use the concept of ‘imagined communities’, and related work on socio-technical imaginaries and expectations, to reflect on how Scotland is represented simultaneously as ‘sick and unhealthy’ and a ‘living lab’ due to its innovative medical research. Together, we suggest these narratives have driven a broader health and wealth agenda in post-devolution Scotland, which became salient during the 2014 independence referendum. We draw on research conducted during the independence referendum to consider how key stakeholders enacted imagined communities/identities (sick but also innovative) as they considered the historical impact of devolution on health and research systems and envisioned future independence. The referendum provided an opportunity to consider how Scottish health and research systems have been imagined over time. Our findings further the understanding of the impact of devolution on healthcare and medical research, revealing the role played by policy narratives rooted in imagined identities.

## Introduction

The delivery of Scottish healthcare through the devolved National Health Service (NHS) is more politicised and publicly salient than the less visible UK research system, to which Scotland contributes its scientific and medical expertise and institutional assets. The symbiotic relationship between healthcare and research is important, but marginalised by political parties and under-analysed by health innovation and policy researchers, although some have explored hidden innovation within specific healthcare contexts (Hopkins, 2006). However, the primacy of the interdependent relationship between healthcare and research is important in the context of recent debates about Scottish devolution and independence, which have become fevered in light of the 2014 referendum decision for Scotland to remain part of the UK and more recent developments around Brexit, which has created uncertainty about the future of the UK and its geopolitical relationships.

Here, we explore how a range of narratives and metaphors around health, wealth, and scientific/clinical research and innovation emerged in post-devolution Scotland and shaped a range of imagined communities or identities. We ask what specific narratives have underpinned Scottish healthcare and medical research systems since devolution, and how were these reflected in speculative and imaginative accounts of the benefits and limitations of independence? To answer, we explore the assumptions around identity and community that underlie the dual narratives of Scotland having a unique set health challenges – most recently expressed as the ‘sick man of Europe’ (McCartney et al., 2011) – and a population demographic that is used to present Scotland as a viable ‘living lab’ for the research community (Smith et al., 2006). Drawing on Anderson’s (1991) ‘imagined communities’, and those who have used it in studies of nationalism (Beland and Lecours, 2008; Bond et al., 2003), as well as related work on sociotechnical imaginaries and expectations, we tease out these narratives and link them to the broader ‘health and wealth’ agenda that became a salient trope and policy driver in post-devolution Scotland.

In the following section, we describe our multi-method approach. Next, we outline the theory of ‘imagined communities’ and socio-technical imaginaries and expectations, which informs our analysis. We also briefly outline the history of Scottish devolution and relationship between the health and research systems to provide context to our study. We then unpack the ‘sick man of Europe’ narrative and the ways in which it framed health policy in Scotland, before exploring the ‘living lab’ narrative to reveal how Scotland’s population was presented as a valuable resource for the clinical research community. We show how together these two narratives have driven a powerful ‘health and wealth agenda’ by both policymakers and the innovation community. We highlight the tensions and continuities in these particular ‘framings’. In discussing these narratives/framings, which operate at different levels, we draw on our data and policy analysis to show how they resonate in both reflections on devolution’s past, and more speculative, future-oriented discussions of an independent Scotland, specifically anxieties around research funding, the geographical boundaries of the research system, and the complex relationship between health and research systems.

## Methods

Data were collected as part of an Economic and Social Research Council (ESRC) funded project, ‘Scottish Independence and Health’, part of its ‘Future of the UK and Scotland’ programme. During 2013/2014, we collated and analysed government and non-governmental strategy reports and white papers relating to healthcare and medical research, to uncover how needs and priorities were identified under a fully devolved healthcare system and partially devolved research system (early 2000s–2014 referendum). We also conducted 15 semi-structured stakeholder interviews – 6 academic/clinical scientists, 3 NHS R&D managers, 3 representatives from funding agencies; and 3 policymakers (individuals who had worked in devolved government agencies on innovation policy). These categories are not fixed as most interviewees had worked across professional boundaries, for example, some academic and clinical scientists had experience/contributed to health and research policy. Some involved in funding agencies also had prior experience in more government and policy-oriented roles. So we do not assume these purposively sampled elite interviews are representative of distinct constituencies and operate as stable categories. Nevertheless, they revealed a range of views on the benefits and limitations of devolution and provided us with speculative accounts about independence that drew on constructed sick and innovative identities and socio-technical imaginaries. The open-ended interview questions focused on the respondents’ views on the idea of Scottish independence, in the context of healthcare and medical research, but all were encouraged to also reflect on the benefits and limitations of devolution.

We also conducted a media search, using Lexis Library of newspaper articles from three Scottish national newspapers – the Scotsman, the Herald, and the Daily Record – in the pre- and post-devolution years (1993–2005) to help us understand how the idea of a unique Scottish health population became popularised. All relevant articles (56 relating to health were selected from a return of over 21,000 from our initial search) were compiled by date and read chronologically to identify relevant themes. This method provided a broader and ‘populist’ context to our data from elite stakeholders.

Finally, we organised a small workshop (12 participants) with a similar group of stakeholders to those we interviewed, but also including representatives from some commercial research organisations. This was organised by the lead author (Mittra) following the interviews. We disseminated initial findings to generate further debate among our workshop participants and identified areas of agreement and contestation on the issues of devolution, independence, and impacts on health and research systems. This refined our findings.

Interviews were recorded and transcribed anonymously (only area of expertise is indicated), and the workshop discussion was recorded and notes taken. Analysis of the interview/workshop data, and policy and media documents, was based on thematic coding of key concepts, such as ‘health and wealth’, ‘sick man of Europe’, ‘research funding’, ‘innovation’, ‘clinical trials’, ‘collaboration’, ‘regulation’, and ‘national priorities/identities’, as well as ‘benefits to’ and ‘limitations of’ devolution and independence. An iterative and grounded approach was used to ensure concept development was consistent with the emerging data.

In addition to the interviews, workshop, and document analysis, Mittra also participated in a public event (over 100 participants) and organised another public engagement event with the co-authors (75 participants) during the referendum. The first was part of the 2014 Edinburgh International Science Festival – ‘Choosing a Healthy Future’ – where Mittra was invited to join a panel of two Members of the Scottish Parliament and a clinician at the National Museum of Scotland to discuss and take audience questions on the potential impact of independence on healthcare in Scotland. Mittra spoke specifically about the medical research system. The second event was part of the Edinburgh International Fringe festival’s ‘Cabaret of Dangerous Ideas’, where we organised a 90-minute session titled provocatively ‘Is Scottish Independence Bad for your Health’? We presented our project findings and answered audience questions. Both events allowed us to disseminate the findings of our research as part of our public engagement work (these events occurred towards the end of the project) and get feedback/additional data from a general audience. This provided a contrast to the narratives of our elite stakeholders, but we add the caveat that these public events were less structured than our interviews and workshop, and the data analysis was not as systematic as for our other methods.

## Background to ‘imagined communities’ and Scottish devolution in the context of health and medical research

The notion of Scotland as an ‘imagined community’ has been expressed in various media and policy documents indicating a ‘have not’ status or a ‘have-but-denied’ (i.e. not able to take advantage of) status (Beland and Lecours, 2008; Bond et al., 2003; Scott and Wright, 2012). The theory of imagined communities is useful for understanding narratives around healthcare policy and research, and how they shape reflections on past, present, and future identities.

Benedict Anderson introduced ‘imagined communities’ in an anthropological spirit to explain the universality of the concept of nationalism and show how national identities are rooted in social imaginaries (Anderson, 1991; Beckert, 2016). Others suggest UK regions are not simply administrative units but reflect historical experiences that make up a cultural image; one based on socio-economic processes that reflect the common history of inhabitants (MacLeod, 1998). Beland and Lecours (2008) argue an important factor in modern Scottish nationalism was Thatcher’s ‘neoliberal policies’, which were perceived as an attack on institutions cherished by the Scottish people. Scottish national identity became associated with notions of communitarianism and social justice (Scott and Wright, 2012). Social policy is often presented as reflective of core values, which fit into the language of nationalism and the range of discursive narratives that underpin it. Scotland’s nationalists viewed social policy as central to Scottish identity, in contrast to Thatcher’s nationalism, rooted in the spirit of enterprise, which had supposedly been diluted by social democracy (Bond et al., 2003: 373). Following post-positivist policy studies by scholars like Fischer (2003), we suggest subjective discourses, narratives, and symbolism frame formal policy and actor’s understanding of the world. The focus on narratives, and the way policy actors reflect on and express their views, allows space for the salient, subjective understandings that contribute to the making of policy and identity.

Health offers a good example of devolution and divergence, in that Scottish health policy distanced itself from English policies by resisting marketisation and consumerism in healthcare, and calling for approaches to meet the specific needs of the Scottish population, as our data reveal. This aligns with the work of Greer (2005), who suggests there are territorial divides throughout the UK on health policy, although in the context of ‘public health policy’, Smith and Hellowell (2012) suggest there is more convergence. Similarly, in his analysis of the four UK health systems, Timmins (2013) shows increasing levels of divergence, particularly between England and the other devolved nations, in terms of structures, management approaches, and relationship between health and social care. However, he suggests that the data do not allow for easy comparative analysis. Nevertheless, Scotland’s putatively communitarian approach to health policy in the post-devolution era was in tension with a parallel industrial policy to build a life sciences sector and situate Scotland as a global leader for medical research. These tensions became salient during the referendum. In speculating on the future of health and research, our respondents drew on various ‘sociotechnical imaginaries’, described by Jasanoff and Kim (2009) as visions of desirable futures, based on common understandings of social order in a context of technoscience, which are institutionally stabilised, as we highlight later.

We do recognise a limitation of Anderson’s imagined communities approach for this analysis. As Beckert (2016) argues, Anderson focuses on the past and the present when discussing the role of imaginaries in the process of nation building. So it is largely historical and focused at the national level. However, we are also interested in imaginaries or constructed narratives that are speculative and future-oriented, and which reflect multiple identities/communities, not just a monolithic national one. Beckert is useful in that he adapts Anderson’s theory by exploring imaginaries of the future, in the context of economic relations, rather than nation building, referring to these as natural and necessary ‘fictional expectations’. In its adapted form, the concept helps us understand our data in the context of past and future narratives that reflect multiple national and sub-national identities. Linking this with sociological work on expectations (Brown and Michael, 2010), we can better understand the part played by visions of technoscience and social order within both collectivised (policy) and individual accounts of the past and future. But first we provide some context to the medical research and healthcare systems of Scotland.

Since devolution (the1998 Scotland Act), the medical research and healthcare systems of Scotland, and its people, have been presented in policy documents as a ‘research asset’. For example, the life sciences strategy adopted by the Scottish Government in 2005, reiterated in 2011 (The Scottish Government, 2005, 2011), was driven by the belief that health and economic benefits emerge from investments in basic medical research, clinical studies, and research infrastructure. The rhetoric was that Scotland has a competitive advantage in medical research and *should* be capable of responding to a unique set of health challenges facing its population, while simultaneously driving economic growth. This alignment of healthcare, innovation, and economic prosperity is part of a more generalised global phenomenon, captured by the changing practices and organisational norms within the now much touted ‘bioeconomy’ (Birch, 2017; Mittra, 2016). Crucial to this emerging health and wealth narrative was Scotland’s well-established disease and research networks; good governance systems and fully electronic patient records; internationally recognised research in life sciences; and, counter-intuitively, a chronically sick population that is relatively stable and localised.

Before we unpack these narratives around health, research, and innovation, it is important to clarify the distinctiveness of the Scottish NHS and its recent history as a fully devolved power, vis a vis the partially devolved medical research system. NHS Scotland was already a separate organisation before devolution, but in 1999 it officially came under the direct control of the Scottish Government. NHS spending is approximately one-third of the total Scottish budget (Robson, 2016). Following changes in the English health system (to a Commissioner and provider model), the Scottish system appears organisationally distinct. The 14 Scottish NHS Boards and 7 Special Boards are all-purpose organisations; they plan, commission, and deliver healthcare. Spending per person – adjusted for age – is slightly higher in Scotland than in England (Robson, 2016). Spending on the baseline infrastructure is devolved to national governments (in Scotland via the Scottish Funding Council), but the research grant system is UK-based (delivered via the UK’s Department for Business, Innovation and Skills (BIS) to Research Councils UK (RCUK)). In health and medical research, this is reinforced by trust and charitable funding, especially the Wellcome Trust. In this unusual situation of fully devolved healthcare (delivered by NHS Scotland), and a partially devolved and more porous research system (linked to broader UK and EU initiatives), a distinct set of narratives around health, research, and innovation emerge.

## Narrative 1: Scotland as the ‘Sick Man’ of Europe

Our first narrative relates to the fact that Scotland has long held the pejorative label of the ‘Sick Man of Europe’. Since the late 1970s, compared to 19 other European countries and the rest of the UK, Scotland has had the highest mortality among working age men and women (Whyte and Ajetunmobi, 2012). In 2009, mortality for young Scottish males was 54% higher than the rest of the UK. The city of Glasgow suffers poorer health outcomes than similar sized UK cities with comparative levels of social deprivation, known as the ‘Glasgow Effect’. In a study of three UK cities (Manchester, Liverpool, and Glasgow), Walsh et al. (2010) observed:

Premature morality (under 65 years) in Glasgow has been shown to be 30 per cent higher than in the identically deprived UK cities of Liverpool and Manchester, with deaths at all ages almost 15 per cent higher. This ‘excess’ has been shown for all adult age groups, both sexes and across different neighbourhood types (deprived and non-deprived). (p. 8)

Our media analysis revealed from 1993 to 2005 the Scottish print media popularised the narrative of Scots being disproportionately sick and unhealthy. Headlines such as Scotland being ‘the sickest in the UK’ (Daily Record 15 February, 2001) emerged as data revealed that cancer and heart disease accounted for 45% of deaths, with a mortality rate (11.8/1000) worse than many Eastern European countries. The media were keen to emphasise the exceptionalism of Glasgow and its status as being 114 out of 120 authorities on the ‘sick list’, despite receiving 20% more health funding than most English authorities (The Scotsman 21 February, 2000). Similarly, other newspapers suggested that the population was ‘Healthy but not Happy’ (Daily Record 2 March 2000), as stress and depression appeared to be increasing. Other newspapers suggested young people were ‘hooked on sex, chips and cigarettes’ (The Scotsman 18 April, 2000), despite evidence of marginal improvements in survival rates for cancer and heart disease. The Daily Record (25 January 2001) predicted that up to 100,000 Scots might ‘flee by 2020’ due to poor health and lack of jobs.

There was also an attempt to link poor health outcomes in Scotland to growing wealth disparities (Scotsman 18 November, 1995), which is a long-standing historical issue that many scholars have identified and responded to within a broad ‘social determinants of health’ framework (Davidson et al., 2007; Macintyre, 1997; WHO, 2008). Within both popular and professional discourses, the rhetoric of a ‘survival of the richest’ (Herald 21 February, 1997) was salient from 1995 to 2000, contrasting the ‘haves’ and ‘have-nots’. An article in the Herald suggested cancer rates in Scotland reflect the wealth and affluence of an area so precisely that postcodes are used to identify at-risk individuals (Herald 13 February 1995).

This focus on Scotland’s health status and its population as exceptionally unhealthy was also reflected in government policy. A Scottish White Paper on independence (The Scottish Government, 2013) included a section on health, social care, and the NHS. The narrative positively set out what the Scottish National Party (SNP) administration saw as the major benefits of devolution (allowing Scotland to respond to national health needs), and the challenges and opportunities offered by full independence. On health, it reassured the public that access to NHS services would not be negatively affected by a Yes vote, because healthcare is devolved. The advantages of this were set out in the document and drew explicitly on notions of Scottish exceptionalism and the challenge of meeting specific health needs:

Despite efforts to address the challenge of health inequalities in Scotland over recent years, health inequalities persist and demonstrate that the ‘fundamental causes’ of health inequalities – the socio-economic inequalities in society – are the most important. (The Scottish Government, 2013: 173)

The paper argued that independence could facilitate a transformation in the environment within which the NHS operates so that ‘health inequalities’, which are reflected in this sick man of Europe narrative, can be tackled more effectively.

The imagined ‘sick community’, resulting from social and economic disparities, was also a prominent trope in both our media and interview data. This ‘sick man’ narrative, in comparison to our subsequent narratives, is more dependent on other, higher level narratives, because it is so indelibly linked to national Scottish policy in terms of both its implications and viable interventions. Also, it relates to other, sub-narratives around Scottish communitarianism and health (Scotland’s NHS being central to this). For example, a Care Quality Strategy for NHS Scotland 2010 report (The Scottish Government 2010) outlined the broader social and economic benefits of a healthy working population (more people in the workforce, fewer sick days, higher productivity) and the need for an efficient health service that uses performance measures involving patients. It built on a 2007 report: *Better Health: Better Care Action Plan* launched by the Scottish Government (NHS Scotland, 2007), stating:

The Healthcare Quality Strategy will ensure that we maximise the contribution of NHS Scotland to the wider Purpose of the Scottish Government to create sustainable economic growth and opportunities for everyone in Scotland to flourish. (The Scottish Government, 2010)

Here, a narrative of good population health is bound up with promissory expectations (Brown and Michael, 2010) of a healthy and prosperous nation, in contrast to the ‘sick man’ that is holding the country back. The report also contained distinctively Scottish elements, especially in terms of governance, with a focus on policies like alcohol pricing to reduce consumption, and a call for greater involvement of local government in social care, highlighting a sense of Scottish exceptionalism rooted in a social care/justice framework.

In both our public engagement events, the poor health of the Scottish people – in terms of chronic illness caused by social deprivation – and the importance of devolution and potential independence in better responding to Scottish needs and priorities through the NHS, was a concern for our audiences. There was a sense that Scotland’s identity was partly shaped by its poor health outcomes and that a more communitarian approach to healthcare provision, and improved public health policy, should be priorities. For instance, in both events, members of the audience independently raised the need to tackle social deprivation more effectively and questioned why we perhaps focus on high-tech solutions (captured in promissory socio-technical imaginaries) to health problems rather than on social interventions. We will come back to this issue later in the context of the health and wealth agenda. But we now move on to the second, related narrative.

## Narrative 2: Scotland as a ‘living lab’ for medical research

Our interview and workshop data uncovered many factors that contribute to Scotland’s population being seen as a clinical asset or ‘living lab’. Taking the pejorative ‘sick man of Europe’ label, this new narrative uses it to present Scotland as a place to conduct valuable medical research. It is within this context that the important but complex link between the health and research systems becomes salient. An example of the ‘living lab’ narrative can be found on the Health Sciences Scotland website, which presents ‘high rates of complex diseases’ as a ‘unique resource’ and ‘opportunity’ for health researchers. Our interview respondents and workshop participants involved in drug development suggested that disease burden in Scotland can enable development of more effective treatments to improve public health and deliver economic benefit. Various arguments are invoked to support this living lab narrative.

First, the Scottish population is presented as relatively stable and homogeneous (necessary for certain kinds of genetic studies) and willing to participate in research to improve public health. The latter reflects the supposed altruistic spirit within Scottish identity, which is key to this imagined community. Second, it is a population where all the major chronic diseases are well-represented, as reflected by the ‘sick man’ narrative. Third, Scotland has strong research capacity, medical record linkage, disease registers, and tissue banking facilities, so there is a narrative here about innovation both in terms of science and organisational/institutional assemblages. Finally, Scotland has health boards, governance, and approval systems that are – according to our interview respondents and reflected in numerous policy documents since devolution – more streamlined and efficient than the rest of the UK. As one Scottish NHS R&D director put it,

Scotland works very well together in a way that England doesn’t and probably can’t … All the health boards in Scotland have grouped together and we have now come together as NHS Research Scotland. So we’re now pulling our weight as the whole country rather than individual health boards. (Senior NHS R&D Manager 1)

This national infrastructure for a living lab in Scotland is advanced and driven various projects, including ‘Generation Scotland: The Scottish Family Health Study’ – a population based research study involving the genetic identification of complex diseases, such as cancer, heart disease and mental health, through the recruitment of 50,000 family individuals. A senior academic involved in this study explained that Scotland had the population demographic for such an ambitious project, which was built on promissory expectations that genomics research could transform future healthcare. This ‘unique’ population was used strategically in the funding bid:

I think there was definitely a strong Scottish identity in Generation Scotland, which, I don’t know if it brought us together, but it certainly was what glued us together as we were writing the application and more particularly when we were presenting it to potential participants, and publics and media. (Senior Academic Scientist 1)

Here, we see an explicit account of the tangible benefits of a living lab narrative, and the strategic use of ‘national identity’ to support a prospective research project that values the Scottish population as a research asset.

A number of Scottish White Papers and policy strategies (Scottish Office, 1991, 1993, 1997) addressing major health challenges have also emphasised, at least implicitly, both the ‘sick man’ and ‘living lab’ narratives (The Scottish Government, 1999). In linking medical research investment to meeting the health needs of the Scottish people, policy solutions to public healthcare challenges reified Scottish exceptionalism. The living lab narrative, which aligns investments in research and innovation with improvements in both health and economic development, is rooted in a presumption that healthcare and medical research are connected, although their institutional and geographical linkages and drivers are quite different. Many interviewees spoke of a positive link between the maintenance of a strong medical research system and the quality of healthcare delivered to local patients in hospitals where research is located. One clinician stated,

… there’s lots of observational data … factors of 10:1 are generally quoted of the value that accrues to health systems by having research going on. Patients get better care that are taking part in research and newer, better things get brought into health systems fast as a result of research, you get more skilled workers because research is going on rather than it just being a service commitment (Senior Academic Clinician 4).

Another respondent suggested those regions with significant research capacity attract the best doctors and nurses, who not only drive the research agenda but also deliver quality healthcare to patients. This narrative about the public health benefits of basic clinical research suggests that policymakers ignore the symbiotic relationship between healthcare and medical research at their peril. One workshop participant (a clinician in the NHS) argued that patients who do not have access to a university hospital are ‘materially disadvantaged’.

This enduring link between research and healthcare was marginalised during the referendum and was not a salient theme among members of the public at our events. Nevertheless, it has implications for the funding of research and the scale and scope of the geographical research area, which was a significant concern during the referendum debates. Here, different actors and institutions speculated about the future of Scottish medical research, and the imagined innovative Scottish community. This theme most illustrated the power of speculative imaginaries and the role of hope, expectation, and concern in the context of growing uncertainties (Brown and Michael, 2010).

Continued funding for Scottish medical research was a concern expressed by academic scientists and clinicians, driven by fear they would lose access to RCUK funding under independence (Gibney, 2014). An open letter from the Academy of Medical Sciences, the British Academy, and the Royal Society represented the institutional perspective when it stated,

Research requires resources, permeability, interactions, critical mass and a highly skilled workforce, to drive improvements in the quality of lives and a modern knowledge-based economy. Scotland has long done particularly well through its access to UK research funding. If it turns out that an independent Scotland has to form its own science and research budget, maintaining these levels of research spending would cost the Scottish taxpayer significantly more. (https://www.britac.ac.uk/sites/default/files/British%20Academy%2C%20Royal%20Society%2C%20Academy%20of%20Medical%20Sciences%20Scotland%20letter%20July%202014.pdf)

Here, the porous and institutionally complex research system (in terms of geographical boundaries), which has benefitted Scotland, is presented as at-risk from independence. The letter proceeds to state that strong links and collaborations that have been established under the current ‘open innovation system’ would be under threat. However, not all support for medical research is provided by government. There is a large and influential charitable sector, which during the referendum considered how its operational strategies might be impacted. The dual narratives of the ‘sick nation’ and ‘living lab’, which emerged under devolution, framed these speculative accounts about an uncertain future for research.

Our interviewees from major charities stated that their mission is to fund the ‘best science’. They also claimed that they spend more in Scotland than they raise in Scotland, so the narrative of Scotland being a good place to do medical research and conduct clinical trials (making it a viable living lab) is not hype. It has a material impact on company and charitable investment and national research policy. However, some charitable funders suggested that if Scotland left the UK, they would have to treat it like other non-UK countries. Their view aligned with that of the Wellcome Trust (2013), which released a statement that its eligibility criteria for funding Scottish institutions would be reviewed if Scotland became independent. However, our respondents from charities felt the best science should be funded, and as clinical research crosses borders, it should be possible, though not straightforward, to fund arrangements in an independent Scotland. One stated,

Where it would get tricky for a charity like us, which is a national charity where we’ve got an office in Scotland, and where it would be a disservice to people and clinical research [would be] if you started to have say Prostate Cancer UK becoming Prostate Cancer Scotland, Diabetes UK becoming Diabetes Scotland, … all of these UK charities becoming homed in Scotland and only doing work in Scotland. (Director of Research, Small UK Medical Charity)

Here, we have a speculative ‘problem narrative’ attached to independence; that narrow parochialism, rooted in a distinct narrative of an exceptional Scotland, would emerge if spending became geographically circumscribed. The respondent continued to state that the servicing of committees and grant rounds is already expensive, so it would be inefficient to be restrictive on where money is spent. Others suggested charities generally follow RCUK priorities and there would be concern if independence changed national prioritisation and the way research councils worked together:

If the organisations in the independent Scotland were aligned operationally with the rest of the UK, if the priorities worked well in terms of research prioritisation and the funding of infrastructure was aligned, and if funding support were maintained [government top up to charitable funding of direct costs] in an independent Scotland in a consistent way then I don’t think charities would find it an issue. But all those things are uncertain. (Chief Executive of major charity)

This uncertainty meant it was not clear to charities what would be administratively possible or desirable in terms of continued support for medical research. Also, as other respondents stated, charitable funding is mobile and could move out of the country quickly, because it does not fund infrastructure and indirect costs. Here, respondents were envisioning a potential threat to the perceived status of Scotland as a world leader in medical research, which underpins the living lab narrative, if it was not part of a UK-wide research area.

Our interviewees and workshop participants speculated that it would be disadvantageous to reduce the scale of UK research and lock Scotland out of competitive funding steams and were confident solutions would be found. One UK funding agency respondent said,

… you could imagine a system where the main funding mechanisms of the research councils were open to Scotland and Scotland participated in them but there were various strategic schemes where Scotland has to either opt in or opt out and say we’ll do something differently ourselves. It would be complicated but I don’t think it would be impossible. (Senior Representative of Funding Agency 1)

Here, the complexities of national research priorities and the meeting of research expectations under independence are presented as manageable, so long as there are agreements to maintain the integrity of a single research area. This would benefit the UK as well as Scotland, as large competitive research systems are, according to this respondent; ‘generally better and more efficient’ in delivering excellence. He again used the narrative of competition raising standards, arguing that as research systems get smaller they become less competitive. Size and international scope supports the maintenance of an imagined innovative research community.

However, this view does not sit easily with healthcare system narratives and communitarian ideals that have provided support for a fully devolved health service to meet Scotland’s needs. Other respondents suggested that being part of a smaller research system, rather than an institutionally and organisationally complex UK-wide system, might enable strategic focus and differentiation from the rest of the UK, to better satisfy Scottish health needs. This again raises the issue of Scottish exceptionalism in terms of its public health status. One respondent stated,

… in the past we’ve always kind of followed what England has done so in some ways it would be nice to have those shackles removed and actually get the freedom to say, right, lets really put in place what’s good for Scotland and Scotland’s patients. (NHS R&D Director)

Here, the benefits provided by fully devolved healthcare are seen as potentially benefitting a Scottish-centred innovation system more clearly aligned in terms of focus and priorities. Scientists were divided on whether full independence would be good or bad for Scottish research, but from a public funding perspective, most respondents suggested it would make sense for an independent Scotland and the rest of the UK to continue sharing the infrastructure for competitive funding and maintain a single research area. The institutional and research cultures of the countries that comprise the UK are not particularly different (see again, Smith and Hellowell, 2012), and most respondents felt should be capable of working together for the benefit of all. Interestingly, the notion of a single research area also had implications for commercial investment in clinical research. Our commercially oriented workshop participants revealed that major pharmaceutical and medical device companies want to invest in a fairly homogeneous system, so when research area and funding mechanisms become too regionalised investment strategies are affected. Again, the narrative of scale and scope in the context of competitiveness is prominent in the speculative imaginings of this set of stakeholders.

So far, we have highlighted two different, but related, narratives that have become salient since devolution, and reflect different aspects of Scottish identity and community. The first reflects a Scottish exceptionalism for which a more communitarian approach to healthcare delivery and policy is presented as having a positive impact. Here, the fully devolved health service that has avoided privatisation becomes emblematic of Scottish social justice, supported by broader government policy to tackle social deprivation. The second reflects a more global innovation-oriented narrative, where Scotland’s strength in medical research presents an opportunity to treat the ‘sick nation’ as a living lab. Although the research system is more closely connected with broader UK and international innovation systems (it is not fully devolved like health), it has been co-opted to highlight the benefits of research investment to public health. However, there are tensions between these two narratives, and these become clearer when we consider the emergence, since devolution, of a powerful ‘health and wealth’ agenda underpinning national and regional policy. Through unpacking this agenda, we can further explain the emergence of different imagined communities/identities in post-devolution Scotland, and how they framed speculative discussions around independence.

## The ‘health and wealth agenda’ and Scottish exceptionalism

Since devolution, the linking of public health, medical research, and economic benefit has been a recurrent theme in Scottish policy narratives on innovation and healthcare. It has also framed ‘fictional expectations’ (Beckert, 2016) about the benefits and limitations of independence. Scotland’s 2011 Life Sciences Strategy Report stated,

Our ambition is to double the economic contribution of Life Sciences to the Scottish economy by 2020. Part of the future vision presented is that the National Health Service (NHS) moves centre stage as a key customer for Scottish Life Sciences businesses and a pivotal stimulator of innovative products and services. (The Scottish Government, 2011: 3)

Here, three key elements drive policy – health, research, and economic impact/benefit. NHS Scotland represents the health delivery aspect. As discussed, it is relatively autonomous and able to organise and deliver services in line with Scottish health needs. Within the NHS, research has traditionally been marginalised. The Scottish NHS Chief Executive’s Annual Report (NHS Scotland, 2012) does not mention basic research, but prioritises patient waiting times, delivery of key services, and patient-related outcomes. Here is a prioritisation of the need to deliver effective healthcare (drawing on the ‘sick man’ narrative) and a marginalisation of the ‘living lab’ narrative that has been central to the research and innovation agenda. When research is mentioned in the report, it concerns downstream and applied activities such as assisted living to improve home care. This narrative of care is about unlocking new markets in social innovation, which is far from the high-tech medical research that Scotland markets to international collaborators and investors as part of its national health and wealth strategy.

Nevertheless, the health and wealth narrative has been prominent in the evolution of the Scottish Life Science Strategy to support a small- and medium-sized company sector as part of an evolving biotechnology cluster and national innovation ecosystem (Rosiello, 2007). The 2011 Life Science Strategy took this further by seeking to build innovation capacity and better integrate complementary clinical/medical research assets and healthcare infrastructure, with NHS Research Scotland (NRS) central. Our interview and workshop data revealed a number of contrasting views. Most important for our understanding of ‘imagined communities’ was the perceived desire by many in Scotland to avoid a neoliberal, marketisation of healthcare, and the shared belief that Scotland’s health system was more ‘joined up’ and ‘efficient’ due to its smaller scale and scope.

In terms of healthcare organisation, respondents argued that devolution had allowed Scotland to avoid the major NHS restructuring that has taken place in the rest of the UK and retain a communitarian approach that was a defining feature of the NHS at its inception. This has had a material impact on national policy, which recognises social inequalities and determinants of health, and aligns with the social justice argument that Bond et al. (2003) and Scott and Wright (2012) proposed in their accounts of Scottish exceptionalism. The Scottish White Paper also highlighted the benefits of Scotland’s avoidance of the organisational restructuring of the NHS in England. This reflects an antipathy towards neoliberalism that appears core to this imagined community or identity. As Timmins (2013) notes in his analysis of the UK’s four health systems, Scotland has basically been able to abolish all ‘vestiges of the “internal market”’ (p. 4). Devolution expedited the changes in organisational structure and policy to ostensibly meet the perceived needs of Scotland and, crucially, allow NHS Scotland a degree of protection from more radical reforms. One of our respondents said this has: ‘helped us to maintain productivity within the healthcare sector that we had before simply because we haven’t been party to the large experiment that’s taking place south of the border’(Senior Clinical Academic 2). A stable and joined-up healthcare infrastructure is presented here as a key benefit of devolution.

The issue of commercial versus non-commercial clinical research within the NHS is important in this context, revealing tension in the relationship between health and wealth. Our interview data revealed different views about the benefits and limitations of commercial involvement in a public health service.

From an economic point of view, clinical resources are an asset that can promote collaborative activities within Scotland and with UK and international public and commercial partners. Linking commercial investment and public research assets to strengthen the overall health innovation ecosystem was captured in all of Scotland’s Life Science Strategy documents. However, in practice, there have been few commercial clinical studies within Scotland, or private provision within Scottish NHS. Timmins suggests this is because there is a much smaller commercial sector in Scotland and government policy has purposely avoided reliance on it. Some of our interviewees argued commercial research is marginalised within the healthcare system because it has low status:

… there is a thought out there with consultants that commercial studies are somehow lesser than academic studies … There is definitely a cynical attitude in the NHS towards commercial research. (Senior Clinical Academic 3)

The living lab, and emerging socio-technical imaginaries about improving broad public health through advanced medical research, is essentially built on the development of non-commercial clinical activities, of which the aforementioned Generation Scotland is a good example (Smith et al., 2006).

For some of our respondents, commercially driven research was considered unimportant. In their imagined Scottish health community, the dominant narrative was the need to prioritise healthcare delivery and support publicly oriented research that met local population needs. Wealth creation was secondary. Two of our NHS respondents argued that Scotland should focus on diseases where poverty and social deprivation are the major causes, or perhaps in rare conditions where Scotland has a disproportionate population, such as multiple sclerosis and motor neuron disease. Here, non-commercial studies have real clinical and social value. Others felt Scotland should exploit commercial research more aggressively and decried the negative cultural attitude towards commercial research within the NHS, as earlier quotations highlighted. They also expressed concern that if research became too niche and regional, it would threaten Scotland’s status as a world leader in life sciences. Our public engagement events were revealing in that participants had limited interest/knowledge of this medical research system and the role commercial research plays. A vocal minority expressed concern about commercial involvement in NHS activities; viewing this as part of the broader neoliberal agenda, again revealing the imagined Scottish community underpinned by an identity rooted in social justice.

However, accounts which prioritise either investments in medical research (including commercial studies) or basic healthcare delivery to respond to the ‘sick man’ label fail to recognise the interconnectedness of the healthcare and medical research systems, as discussed earlier; a point which is crucial to any speculative discussion about the benefits and limitations of independence to both health and research.

## Conclusion

Scottish research and healthcare systems have undergone profound structural and organisational change since devolution, and the complexities of how these might evolve under independence animated debates. Questions were raised about Scotland’s capacity and future priorities under independence, with interview respondents, and the narratives embedded in key policy documents, drawing on or reflecting various tropes around Scottish exceptionalism, health and wealth, and the living lab, as different futures were envisioned and potential benefits and limitations articulated.

The existence of multiple and competing narratives about the past and the future of Scotland has implications for viewing Scotland as a set of imagined communities/identities, in this case ‘sick’ but also ‘innovative’, which are problematised within a broader health and wealth policy agenda. Our research revealed mixed views in terms of what the benefits and limitations of independence might be, and how successful devolution had been in shaping the health and research systems. The outstanding questions are how might policymakers imagine innovative research translated into reduced health inequalities both within Scotland and the rest of the UK and, more broadly, how might health and wealth agendas be ultimately reconciled? Understanding the ways in which our respondents’ views were framed in the context of a set of populist narratives that have become embedded in post-devolution Scotland help us better understand the complex and evolving relationship between health and research systems within Scotland and throughout the UK.
